# Concept Recognition and Characterization of Patients Undergoing Resection of Vestibular Schwannoma Using Natural Language Processing

**DOI:** 10.1055/s-0044-1786738

**Published:** 2024-05-11

**Authors:** Simon C. Williams, Kawsar Noor, Siddharth Sinha, Richard J.B. Dobson, Thomas Searle, Jonathan P. Funnell, John G. Hanrahan, William R. Muirhead, Neil Kitchen, Hala Kanona, Sherif Khalil, Shakeel R. Saeed, Hani J. Marcus, Patrick Grover

**Affiliations:** 1 Wellcome/EPSRC Centre for Interventional and Surgical Sciences, University College London, London, United Kingdom; 2 Victor Horsley Department of Neurosurgery, National Hospital for Neurology and Neurosurgery, London, United Kingdom; 3 Department of Computer Science, Institute for Health Informatics, University College London, London, United Kingdom; 4 Department of Biostatistics and Health Informatics, NIHR Biomedical Research Centre, University College London Hospitals NHS Foundation Trust, London, United Kingdom; 5 Department of Informatics, NIHR Biomedical Research Centre, South London and Maudsley NHS Foundation Trust and King's College London, London, United Kingdom; 6 Department of Biostatistics and Health Informatics, Institute of Psychiatry, Psychology and Neuroscience, King's College London, London, United Kingdom; 7 Ear Nose and Throat Department, The Royal National ENT and Eastman Dental Hospital, University College London Hospitals, London, United Kingdom; 8 University College London Ear Institute, London, United Kingdom

**Keywords:** artificial intelligence, vestibular schwannoma, machine learning, Natural Language Processing, retrosigmoid, translabyrinthine

## Abstract

**Background** Natural language processing (NLP), a subset of artificial intelligence (AI), aims to decipher unstructured human language. This study showcases NLP's application in surgical health care, focusing on vestibular schwannoma (VS). By employing an NLP platform, we identify prevalent text concepts in VS patients' electronic health care records (EHRs), creating concept panels covering symptomatology, comorbidities, and management. Through a case study, we illustrate NLP's potential in predicting postoperative cerebrospinal fluid (CSF) leaks.

**Methods** An NLP model analyzed EHRs of surgically managed VS patients from 2008 to 2018 in a single center. The model underwent unsupervised (trained on one million documents from EHR) and supervised (300 documents annotated in duplicate) learning phases, extracting text concepts and generating concept panels related to symptoms, comorbidities, and management. Statistical analysis correlated concept occurrences with postoperative complications, notably CSF leaks.

**Results** Analysis included 292 patients' records, yielding 6,901 unique concepts and 360,929 occurrences. Concept panels highlighted key associations with postoperative CSF leaks, including “antibiotics,” “sepsis,” and “intensive care unit admission.” The NLP model demonstrated high accuracy (precision 0.92, recall 0.96, macro F1 0.93).

**Conclusion** Our NLP model effectively extracted concepts from VS patients' EHRs, facilitating personalized concept panels with diverse applications. NLP shows promise in surgical settings, aiding in early diagnosis, complication prediction, and patient care. Further validation of NLP's predictive capabilities is warranted.

## Introduction

Artificial intelligence (AI) offers novel solutions to surgical problems. The transition of modern medical records from paper to electronic health records (EHR) has generated an abundance of data that remains largely untapped and presents significant opportunities in patient care, diagnostics, administration, and research. 1 However, up to 80% of patient information resides in unstructured text entries. 2 Natural language processing (NLP) is a branch of AI concerned with computer understanding of human language. 3 In a health care context this relates to interpreting and contextualizing text data in the EHR such as clinic letters, narrative documentation, investigation reports, and operation notes. 3 4 In this study, we aim to demonstrate how NLP could be applied in surgical health care settings, using vestibular schwannoma (VS) as an exemplar. VS is both rare (with a reported incidence of 10.4 per million per year 5 ) and complex (typically managed in dedicated skull base neurosurgical units). These factors make the diagnosis of VS challenging, resulting in diagnostic delay. 6 This complexity and unfamiliar relationship that most doctors have with VS makes it an ideal exemplar for AI assistance, in this instance NLP.

This study explores the use of an NLP platform “CogStack.” In simple terms, CogStack is an AI model that scans medical records (such as ward round entries, operation notes, and imaging reports) and extracts the meaning of the written words. CogStack can review each word and ascribe the true meaning of each “concept.” In Layman's terms, a “concept” can be thought of as the true meaning of a word or sentence. This ability to recognize and link clinical concepts helps standardize the extraction of relevant information from diverse health care provider documentation. NLP applications, such as CogStack, can rapidly process large volumes of clinical data and extract concepts associated with a disease process to create a unique fingerprint of text concepts associated with a disease, complication, or outcome. 7 For example, our group have substantiated the efficacy of NLP in identifying concepts associated with normal pressure hydrocephalus, which may in future aid timely diagnosis and discrimination from mimics. 8

This study aims to identify text concepts associated with surgically managed VS using an NLP model (CogStack) through analysis of free-text clinical data from patient EHRs, to create a unique concept panel associated with surgically managed VS. Resultantly, we aim to highlight how NLP may be applied in a surgical health care setting using VS as an exemplar. Finally, through worked example, we aim to demonstrate how NLP may be used to predict postoperative cerebrospinal fluid (CSF) leak in VS patients.

## Methods

This study presents an analysis of patient EHRs using the NLP model CogStack to identify text “concepts” associated with surgically managed VS, enabling analysis of the number of concept “occurrences.” Here, a “concept” can be defined as the true meaning of the word or sentence, mapped to any medical terminology present in the externally created Systematized Nomenclature of Medicine Clinical Terms (SNOMED-CT) database. For example, “operation,” “amoxicillin,” or “physiotherapy” are concepts that may be extracted. An “occurrence” indicates the frequency with which a particular concept appears in the EHRs of the patients.

**Fig. 1:**
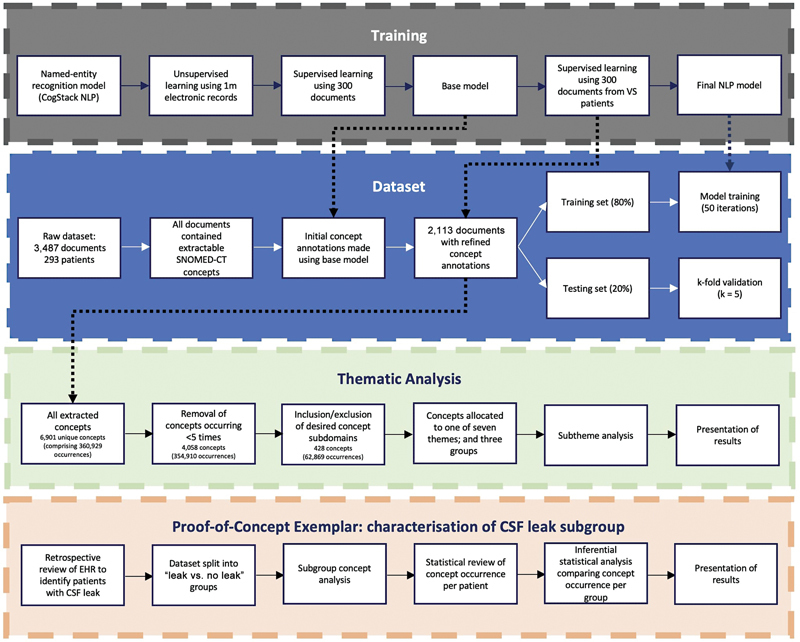
Methodology overview involving in NLP model training, dataset extraction, thematic analysis, and subgroup characterization. Adapted from Funnell et al. 8 CSF, cerebrospinal fluid; EHR, electronic health record; SNOMED-CT, Systematized Nomenclature of Medicine Clinical Terms; NLP, natural language processing; VS, vestibular schwannoma.

Study flow methodology is shown in Fig. 1 . Our methodology is published in accordance with MINIMAR (MINimum Information for Medical AI Reporting) guidelines 9 and represents an IDEAL Stage 0 study in accordance with the IDEAL framework. 10 This study was registered as a service evaluation within University College London Hospitals and was approved by the Local Clinical Governance Committee.

### Patient Selection

Patients were identified through a prospectively maintained database of patients with surgically managed VS at a tertiary academic neurosurgical center in the United Kingdom. Inclusion criteria were patients who underwent primary surgical resection of VS, with histological confirmation of VS, within the 10-year period of 2009 to 2018. Exclusion criteria included schwannomas originating from cranial nerves other than the vestibulocochlear nerve (e.g., trigeminal, facial, peripheral, and spinal schwannomas), neurofibromatosis 2 patients, patients who underwent primary stereotactic radiosurgery, and incomplete medical records. Incomplete medical records were defined as records deemed by the research team to have such substantial areas of missing information that key aspects of VS patient care could not be reviewed (e.g., cases where operation notes or follow-up documentation had not been imported to the EHR).

### CogStack Model and training of MedCAT Platform

CogStack is an information retrieval AI model with NLP facilities that is able to extract written clinical information from data systems (such as health care records) and provide analysis of unstructured text. 11 This model was used for analysis of our VS patient dataset.

Data analyzed included all narrative text-based data, including but not limited to clinic letters, narrative documentation (including ward round entries, nursing, and therapies documentation), investigation reports, and operation notes. No restrictions were placed on the time to and from the operation date.

Documents were mined from EHRs (Epic Caboodle, Epic Systems, Verona, Wisconsin, United States) by the information retrieval model (CogStack). Extracted data then underwent analysis by a machine learning (ML) model (MedCAT) in two phases. The model used is derived from previous work conducted on capturing symptoms for patients with Normal Pressure Hydrocephalus. 8 We describe the model training process below.

In the first phase, an unsupervised named entity recognition and linking (NER + L) ML model was trained using one million EHRs randomly sampled from the Trust-wide EHR. This model was then used to identify terms, synonyms, and concepts linked to SNOMED-CT clinical concepts within the free text. The platform recognized embedded and hidden concepts and generated a list of associated clinical concepts. The ML model then underwent a process of supervised learning refinement, through which 300 written documents were independently annotated with SNOMED-CT concepts in duplicate.

In the second phase, the model underwent further refinement and tailoring toward VS specific pathology. In this phase, a supervised learning approach was taken in which the model annotated 300 documents from our VS patient dataset, which were then validated by two independent assessors in a blinded fashion, using the MedCATTrainer interface. 12 Assessors reviewed the concept labels annotated by the MedCAT platform to determine if they matched the intended meaning of the written text. Concepts were labeled as correct, incorrect, or terminated (for letter artifacts, e.g., names or addresses). Following this initial round of independent annotations, we recorded an interannotator agreement score using Cohen's kappa score. Inconsistencies between the assessors' judgements were resolved through discussion of contradictory concepts between assessors. Any further disagreements were resolved through discussion with a third, independent arbitrator. The validated dataset generated through the MedCATTrainer was used to train the ML model using 50 iterations on a training set accounting for 80% of the extracted documents.

### Concept Extraction

Twenty percent of extracted documents were used as the test set. Accuracy of the model was assessed using k-fold validation (k = 5). Precision and recall were assessed by calculation of a macro f1-score, where the macro F1-score was defined as the average F1-score for all SNOMED-CT concepts identified, and f1-score defined as the harmonic mean of precision and recall.

### Thematic Analysis

SNOMED-CT concepts were extracted from patient records. Concepts with fewer than five occurrences were excluded from analysis. An “occurrence” indicates the frequency with which a particular concept appears in the EHR of the patients. Extracted concepts were grouped by the platform into 59 subdomains, e.g., “clinical drugs” (T-9) and “social concepts” (T-50). Concepts from each subdomain were manually reviewed in duplicate by two authors (S.C.W. and S.S.) for relevance to VS pathology and inclusion in the final analysis. Any disagreements were arbitrated by the senior author (H.J.M.). Concept subdomains included for analysis are shown in Table 1 .

**Table 1:** Concept subdomains selected for analysis

Concept subdomain	Example concepts
Disorders (T-11)	“Acoustic Neuroma,” “Hydrocephalus,” “Hypertension”
Environment (T-14)	“Intensive care unit,” “Clinic,” “General Practise”
Findings (T-18)	“Headache,” “Weakness of face muscles,” “Numbness of face,” “Fatigue”
Morphological abnormalities (T-29)	“Neoplasm,” “Schwannoma,” “Surgical wound”
Observable entities (T-33)	“Address,” “Gender,” “Rest,” “Cognitive function,” “Respiratory rate”
Procedure (T-39)	“Patient review,” “MRI,” “Surgical procedure”
Qualifier value (T-42)	“Neurosurgery,” “Review,” “Stable”
Regime/therapy (T-45)	“Surveillance,” “Rehabilitation therapy,” “Palliative care”
Substance (T-55)	“Drug or medicament,” “Cerebrospinal fluid,” “Ceftriaxone”

Concepts deemed unrelated to VS were excluded from analysis. Concepts were subsequently thematically analyzed to enable grouping into themes. Concepts were assigned to one of three groups, “Signs, Symptoms, and Co-morbidities,” “Inpatient Management, and “Outpatient Management and Follow Up.” Furthermore, concepts were grouped into subthemes, including “signs and symptoms,” “comorbidities,” “demographics,” “patient pathway/event,” “radiological features,” “management,” and “complication.”

### Proof-of-Concept: Concept Characterization of Cerebrospinal Fluid Leak Patients

Patients who experienced postoperative CSF leak were identified through manual review of EHR. Concepts were extracted and analyzed for patients who had and had not suffered a CSF leak. For each group, total concept occurrence was divided by the number of patients within each group to generate a concept occurrence per patient metric. Concepts with a notably variant concept occurrence per patient value between the two groups, and those deemed highly relevant to CSF leak, were selected for further statistical analysis. Inferential analysis was performed using a chi-square test for selected concept occurrences between the leak versus no-leak groups. Effect size is reported using odds ratios (OR). *p* -Values of <0.05 were deemed statistically significant.

## Results

### Demographics

A total of 292 patients who underwent VS resection were included for analysis over the 10-year period (2009–2018). Demographic characteristics of included patients can be found in Table 2 .

**Table 2:** Baseline data for vestibular schwannoma patients included in analysis

	All VS ( *n* = 292)	RS ( *n* = 165)	TL ( *n* = 127)
Age (median + IQR)	49 (38–59)	50 (38–63)	48 (39–54)
M:F	160:132 (55%: 45%)	98:67 (59%: 41%)	62:65 (49%: 51%)
Preop tumor volume (cm ^3^ ) (median + IQR)	8.0 (3.4–13.3)	10.4 (6.2–18.4)	3.9 (1.0–8.1)
Postop tumor volume (cm ^3^ ) (median + IQR)	0.05 (0.00–0.79)	0.17 (0.00–1.14)	0.00 (0.00–0.51)
% Resection (median + IQR)	100% (91–100)	98% (91–100)	100% (91–100)
Presenting symptoms			
Hearing loss	90% (262/292)	85% (140/165)	96% (122/127)
Tinnitus	43% (126/292)	36% (59/165)	53% (67/127)
CN V palsy	22% (64/292)	24% (40/165)	15% (24/165)
CN VII palsy	4% (12/292)	6% (10/165)	1% (2/165)

Abbreviations: CN, cranial nerve; IQR, interquartile range; F, female; M, male; RS, retrosigmoid approach; TL, translabyrinthine approach; VS, vestibular schwannoma.

Note: Regarding MINIMAR (MINimum Information for Medical AI Reporting) guidelines, race, and socioeconomic data were not available.

### Model Training and Evaluation

During the training phase, interannotator agreement for the initial blinded stage concept labeling was scored using Cohen's kappa, with a result of 0.72, indicating substantial agreement. Following this, an additional supervised learning phase was conducted to further enhance annotation accuracy.

Following both the unsupervised and supervised learning phase, the CogStack NLP model was noted to have a precision of 0.92, recall of 0.96, and a macro F1 score of 0.93.

### Concept Extraction and Analysis

A total of 6,901 unique concepts were identified, with 360,929 concept occurrences extracted. Concepts were derived from clinical letters ( *n* = 198,573, 55%) and inpatient notes ( *n* = 162,356, 45%). Fifty-six percent ( *n* = 201,740) of concepts were extracted from clinical notes from patients who underwent a retrosigmoid approach, whereas 44% ( *n* = 159,189) of concepts were extracted from those who underwent a translabyrinthine approach.

Following removal of concepts with fewer than five occurrences, subdomain inclusion/exclusion, and selection of unique concepts deemed relevant to VS pathology, 428 unique concepts, comprising 62,869 occurrences, were included for thematic analysis.

Following thematic analysis, concepts were grouped into “Signs, Symptoms, and Co-morbidities” ( *n* = 20,623), “Inpatient Management” ( *n* = 31,313 concept occurrences), and “Outpatient Management and Follow-Up” ( *n* = 10,933 concept occurrences).

A total of 110 unique concepts were classified as “signs and symptoms,” comprising 10,553 concept occurrences. Selected concepts are shown in Table 3 . Facial weakness was the most commonly occurring concept ( *n* = 801 occurrences) for VS patients, followed by dizziness ( *n* = 492), headache ( *n* = 435), and fatigue ( *n* = 345). Notably, facial numbness ( *n* = 262) and hearing loss/tinnitus ( *n* = 175) did not feature in the top five symptoms/signs.

**Table 3:** Frequency of selected concepts relating to symptoms and signs in the preoperative phase

Sign and symptom theme	Concept frequency
Facial weakness	801
Dizziness	492
Headache	435
Fatigue	345
Pain	264
Facial numbness	262
Hearing loss/tinnitus	175
Nausea	144
Seizure	135

Ninety-four unique concepts were classified as “comorbidities,” occurring a combined total of 7,566 times throughout the EHR. Concepts associated with comorbidities were subdivided into subthemes (e.g., cardiovascular, respiratory), the frequency of which can be found in Table 4 . Neurosurgical comorbidities, including VS, were the most commonly occurring theme ( *n* = 3,826 concept occurrences), followed by neurological comorbidities ( *n* = 949), infectious disease including coronavirus disease 2019 (COVID-19; *n* = 834), and cardiovascular ( *n* = 826).

**Table 4:** Frequency of selected concepts extracted for themes relating to comorbidities

Comorbidity theme	Concept frequency
Neurosurgical	3,826
Neurological	949
Infectious disease (including COVID-19)	834
Cardiovascular	826
Ophthalmic	257
Musculoskeletal	208
Ear, nose, and throat	180
Systemic/multisystem	153
Respiratory	139
Psychiatric	45
Renal/urological	40
Gastrointestinal	30
Diabetes	29
Dermatological	21
Hematological	15
Hepatopancreaticobiliary	14

The “Outpatient Management and Follow Up” concept group contained 47 unique concepts. The most frequent concepts associated with this group were concepts relating to outpatient clinics ( *n* = 6,717; Table 5 ). Stereotactic radiosurgery ( *n* = 1,274) and active surveillance ( *n* = 932) were commonly occurring concepts, making up key components of postsurgical VS management.

**Table 5:** Frequency of selected concepts extracted for outpatient management and follow-up

Concept theme	Concept frequency
Clinic	6,717
Stereotactic radiosurgery	1,274
Therapies (occupational therapy/physiotherapy)	1,127
Active surveillance	932
Botulinum toxin	252
Multidisciplinary team	77
Ophthalmology input	68
Palliative care	34

### Proof-of-Concept: Characterizing Cerebrospinal Fluid Leak

**Fig. 2:**
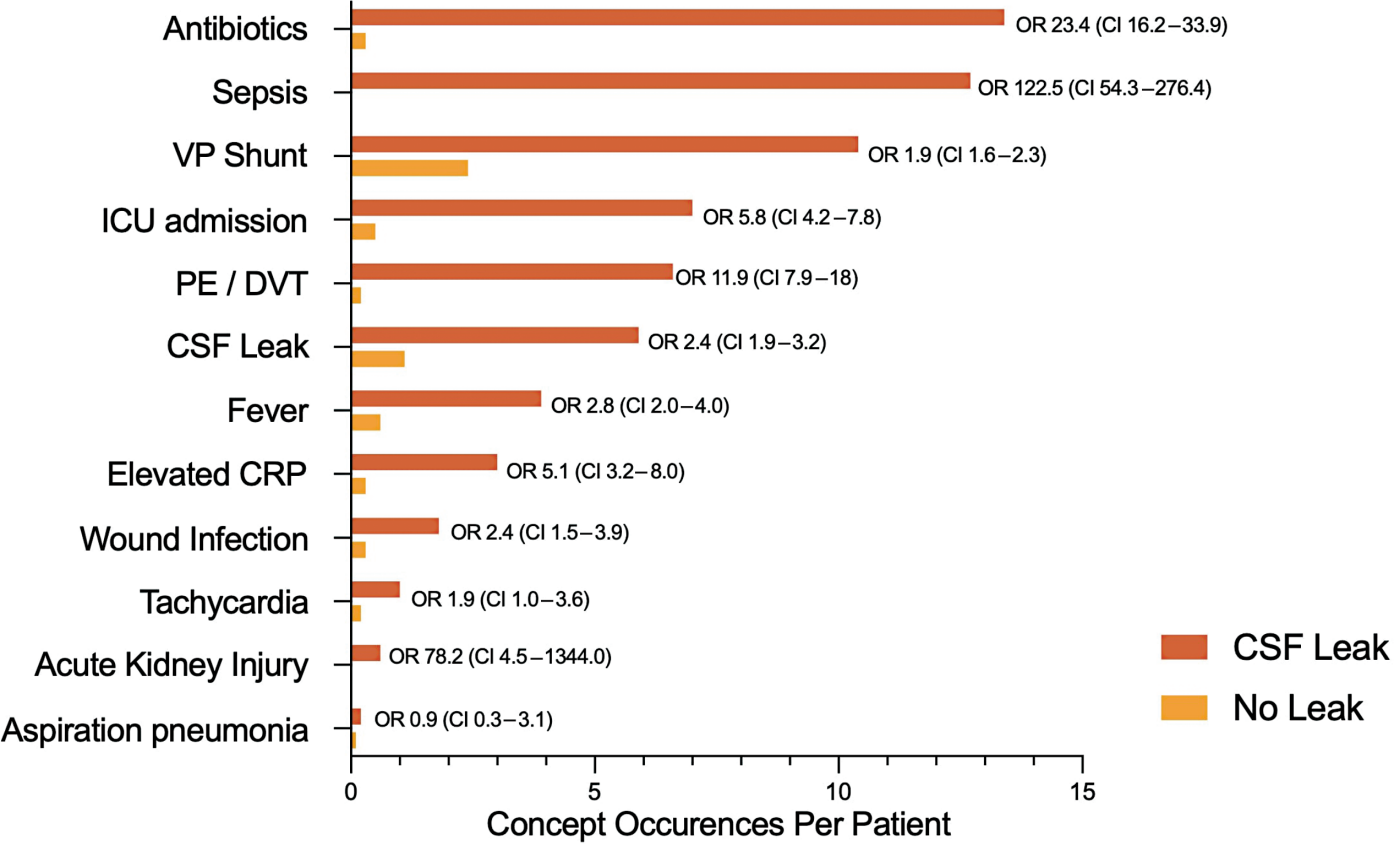
Comparison of concept occurrence per patient between patients who suffered a postoperative CSF leak versus those who did not. A chi-squared comparative analysis was performed comparing the retrospective occurrence of concepts between the leak ( *n* = 25) versus no-leak group ( *n* = 267). Odds ratios and confidence intervals are displayed adjacent to each concept. Numeric figures are presented to one decimal place. CI, confidence interval; CRP, C-reactive protein; CSF, cerebrospinal fluid; ICU, intensive care unit; OR, odds ratio; PE/DVT, pulmonary embolism/deep vein thrombosis; VP shunt, ventriculoperitoneal shunt.

Of 292 patients undergoing VS resection, 25 experienced postoperative CSF leak (8.6%). The concept “CSF leak” occurred at a high rate in our CSF leak group (5.9 occurrences per patient) when compared with the nonleak group (1.1 occurrences per patient; OR: 2.4 [confidence interval: 1.9–3.2]; *p* < 0.05). The concepts antibiotics (OR = 23.4 [16.2–33.9]; *p* < 0.05), sepsis (OR = 122.5 [54.3–276.4]; *p* < 0.05), wound infection (OR = 2.4 [1.5–3/9]; *p* < 0.05), and fever (OR = 2.8 [2.0–4.0]; *p* < 0.05) were all statistically significantly more common in the CSF leak group. A comparison of concept occurrence between patients who did versus did not suffer a postoperative CSF leak are demonstrated in Fig. 2 . Concepts associated with complications not directly related to CSF leak were also significantly more common in the CSF leak group, including pulmonary embolism/deep vein thrombosis (PE/DVT) occurrence, acute kidney injury, and aspiration pneumonia.

## Discussion

### Principal Findings

This study showcases the feasibility of employing an NLP model to comprehensively analyze EHR and extract pertinent concepts, resulting in the creation of a unique concept panel tailored specifically for VS patients. By analyzing a vast corpus of EHR text data obtained from 292 surgically managed VS patients, we successfully extracted and examined over 360,000 occurrences of relevant concepts to effectively characterize this specific patient population. This study aimed to demonstrate the utility of NLP in a surgical health care framework, using VS as an exemplar. Our analysis yielded several key findings.

First, we demonstrate the accuracy of CogStack in extracting relevant concepts in a neurosurgical cohort. The model was noted to have a precision of 0.92, recall of 0.96, and a macro F1 score of 0.93.

Second, using this model, we sought to characterize three groups of variables to generate a patient concept panel spanning the management of VS: (1) symptomatology, (2) comorbidities, and (3) outpatient management. Unsurprisingly, the symptomatology concepts occurring most frequently were those associated with VS presentation (facial weakness, dizziness, facial numbness, hearing loss/tinnitus) or associated with typical neurosurgical postoperative complaints (headache, fatigue, pain, nausea). The generation of a bespoke symptomatology concept panel associated with VS demonstrates how further applications of NLP may be recognized and may represent the first step in using NLP to prompt earlier diagnosis. 6 13 Delays enable lesion growth that ultimately results in deterioration of symptoms, restriction of management options, and an association with poor surgical outcomes. 14 15 The underlying reasons behind late diagnosis in VS are varied and necessarily occur prior to first neuroimaging. 6 Barriers include patient factors (time from symptom onset to first medical presentation) and professional factors (time from first medical presentation to neuroimaging confirming the presence of a lesions). Professional delays are caused by myriad factors, including waiting lists for referrals and neuroimaging pathways, logistic inefficiencies, and misdiagnosis. NLP platforms may reduce delays through automated linkage of symptom concepts in a patient's EHR with a database of pathology-specific concept maps, leading to a list of differentials suggested to the clinician. Practically, this may be introduced in the form of a generative AI assistant/toolbar embedded within the EHR. As such, the AI would work in tandem with the clinician to improve patient outcomes.

Primary care physicians stand to benefit most from this, given the vast mine of untapped text data spanning patients' lives present within primary care EHR. Such an intervention could be automatically indexed to validated resources such as NICE CKS or GPNotebook (used by over 30,000 UK registered doctors each year 16 ), thus increasing efficiency. Indeed, NLP platforms have been used to enhance early recognition of psychosis, 17 postoperative wound infection, 18 and rare neurological conditions. 19

The identification of neurosurgical and neurological comorbidities aligned with the expected association of VS with these specialties. However, the presence of comorbidities such as cardiovascular, musculoskeletal, respiratory, and psychiatric conditions is noteworthy. These findings highlight that VS patients require comprehensive medical management beyond the scope of neurosurgery, emphasizing the importance of multidisciplinary care. Of interest, the theme “Infectious Disease (including COVID-19)” was the third most commonly occurring. Our patient cohort underwent their operations from 2009 to 2018, prior to the COVID-19 pandemic. Nearly all patients had clinic follow-up during the COVID pandemic; however, during that time the concept of COVID appeared in nearly every text document submitted to the EHR. This serves as a reminder that the output of NLP platforms is entirely governed by the text input and can bias findings.

Automated comorbidity concept extraction using NLP is highly valuable with a range of potential applications. First, automated concept extraction may enable AI-assisted anesthetic evaluation. For example, extensively comorbid patients may be flagged as high risk and automatically assigned to a high-risk anesthetic clinic. 20 21 Second, use of NLP to characterize the comorbid/frailty state of individuals may assist in operative planning, outcome prediction, and thus decision-making; several publications in recent years have utilized AI to predict outcomes for VS patients. 22 23

Concepts associated with “ *Outpatient Management and Follow Up* ” were representative of the typical VS postoperative journey, the key tenets of which are *active surveillance* through *clinic* and *MDT* follow-up, *therapies* input, and *stereotactic radiosurgery* . A notable omission is the concept of reoperation, likely explained by the low proportion of our patient cohort who underwent reoperation (6.5%, 19/292). The recognition of key concepts seen in the postoperative journey highlights potential applications for NLP platforms in surgical settings. Automated data extraction is one such application. The advent of EHR has brought with it huge opportunity to revolutionize our means of data collection, away from the resource intense, costly, and often inaccurate means of human data collection, toward automated data retrieval. Automated data extraction through NLP application would streamline efforts in research, quality improvement, and national registry data collection.

For our third key finding, we demonstrate the utility of NLP to characterize VS patients who suffered postoperative CSF leak. Through text data extraction, we highlight the significant increase in certain concept usage within the EHR of CSF leak patients. Concepts of interest include expected terms such as *CSF leak* and *ventriculoperitoneal shunt* ; yet the presence of concepts such as *ICU admission* , *antibiotics* , and *sepsis* highlight the significant morbidity associated with CSF leak. CSF leak is the second-most commonly occurring complication following VS resection (rates vary from 2 to 30% 24 ) and frequently necessitates prolonged admissions, medical treatment, and reoperation, while incurring significant health care costs (median cost of CSF leak following VS resection is $50,401). 25 Early detection is therefore imperative in stemming clinical and economic costs.

This study demonstrates how NLP can generate a bespoke concept map with which patient outcomes and complications may be predicted. Mellia et al published a systematic review of NLP in surgery and noted that prediction of postoperative complications was the modal application of NLP in surgery, demonstrating superior performance compared with non-NLP methods, and accounting for approximately half of the literature in the area. 7 Of note, the existing literature has thus far examined nonspecific surgical complications, such as surgical-site infection, DVT, PE, and infection. 7 Our study furthers this work by generating a concept map associated with a neurosurgery-specific complication.

Ultimately, NLP offers a promising approach toward modernizing the EHR, with benefits possible across the spectrum of disease course. Our study demonstrates downstream value by showcasing how NLP is a practical and appliable approach to rapid data extraction from EHR datasets. This proof-of-concept study represents a first step in NLP research as applied to lateral skull base surgery, the applications of which have the potential to advance patient care, diagnostics, outcome prediction, decision-making, and benefit research. 7 Further research must leverage recent advances in NLP and EHRs to create practical, scalable platforms for integration into modern health care systems, as is beginning to occur. 26 Reporting guidelines for NLP-based literature should ensure high-quality and replicable research output, while stakeholders must safeguard against common pitfalls in NLP research, e.g., underrepresentation of marginal populations, overfitting, lack of evidence-based minimal performance requirements, and limited external validation due to heterogeneity in EHR data entry structures. 7 Practically, integration of NLP tools in a cross-platform mechanism will require significant collaboration between EHR providers and will require buy-in from patients and clinicians alike, both of whom have been open to AI integration. 27 28

### Comparisons to the Literature

The past decade has seen a rise in the number of publications aiming to utilize AI in the diagnosis of VS, although have predominantly focused upon image analysis, 29 30 31 32 33 34 decision-making, 22 and outcome prediction. 23 35 36

NLP is increasingly being used in surgical contexts. Campillo-Gimenez et al describe the use of an NLP platform to predict surgical-site infection in postneurosurgical inpatients, demonstrating a recall and precision of 92 and 40%, respectively. 18 Tvardik et al similarly demonstrated the ability of an NLP platform to detect hospital-acquired infections in a range of surgical inpatients. 37 Furthermore, they comment on the potential for NLP platforms to enable between-hospital comparisons for benchmarking needs, thus enabling identification of outlier performance. 37 The concept of NLP to aid decision-making is supported by Wissel et al, who utilized NLP for identification of candidates eligible for resective epilepsy surgery. 38

Finally, Kraljevic et al describe the next-generation of NLP–health care integration, with their Foresight platform. 26 Foresight is a generative model that analyses structured and unstructured patient data to forecast medical concepts, including diagnoses and treatment strategies. At present, their model is in an iterative stage and is not currently used in clinical decision support. 26

## Strengths and Limitations

This study has several strengths. First, our patient cohort was large for a rare pathology such as VS, capturing data for 292 patients across 10 years. Second, our NLP model has undergone numerous rounds of supervised and unsupervised training, both from a large, diverse dataset of one million electronic records, and a bespoke neurosurgical cohort of patients in a supervised learning phase, iteratively improving the model's accuracy. Third, our work followed an established methodology for the application of CogStack to a neurosurgical problem. 8 Further, this research aimed to counter reporting heterogeneity in the literature of NLP applications in health care by adhering to measures of quality published by Mellia et al, as a by-proxy reporting checklist. 7

This study has several limitations. First, our dataset was derived from a single center and analysis was retrospective, increasing the risk of bias and overfitting. Second, data were derived from hospital patient records, meaning that a diagnosis of VS had been made by time of first referral. To holistically investigate the prediagnostic phase of VS patients and contribute to early diagnosis efforts, a more insightful approach would involve scrutinizing primary care records. Finally, an intrinsic limitation of NLP in EHR is the quality of data extraction is dependent upon the quality of data entry—instances of documentation errors, misdiagnoses, or inadequate documentation could potentially curtail the dependability of the outcomes generated.

## Conclusion

This study highlights the potential of an NLP platform, CogStack, in surgical health care using VS patients as an exemplar. The platform demonstrated a high degree of precision and recall. Second, the platform was used to extract and analyze concepts from the EHR of VS patients to create a unique concept map of symptomatology, comorbidities, and postoperative management. Third, we demonstrate the potential for NLP to characterize patients who suffer postoperative complications, such as CSF leak, through the identification of concept instances linked to these complications. This proof-of-concept study represents a first step in NLP research as applied to lateral skull base surgery, the applications of which have the potential to advance patient care, diagnostics, outcome prediction, decision-making, and benefit research. Future research should focus on prospective validation of this predictive tool.
